# Cholera in Modern Times: Experience From a Tertiary Care Centre in South India

**DOI:** 10.7759/cureus.100023

**Published:** 2025-12-24

**Authors:** Ajith Kumar A K, Gousmahammad Myageri, Pooja R Murthy, Venkatesha Gupta K V, Rakshitha Eashwernath, Meesala Bhargavi Lakshmi, Mohammed Rameez K, Arunkumar Namachivayam

**Affiliations:** 1 Critical Care Medicine, Aster Hospital Whitefield, Bengaluru, IND; 2 Microbiology, Aster Hospital Whitefield, Bengaluru, IND; 3 Biostatistics, Bapuji Dental College and Hospital, Davanagere, IND

**Keywords:** acute kidney injury, antibiotic sensitivity pattern, cholera, metabolic acidosis, shock index

## Abstract

Background

Cholera continues to pose significant public health challenges in regions of the world where inadequate sanitation, overcrowding, or humanitarian crises prevail (conflicts and war), manifesting in endemicity. Cholera outbreaks have also been reported, especially in low- and lower-middle-income countries, mostly during rainy seasons and natural disasters like floods, resulting in contamination of water sources and disrupted sanitation.

Cholera is considered endemic in India, though it causes sporadic outbreaks and seasonal epidemics. Yet, the medical literature regarding clinical presentation and microbiologic patterns of this notifiable ancient disease in areas of rapid urbanization and globalisation in India or elsewhere is scant. This retrospective study aims to analyse the clinical and microbiological profile of patients with diagnosed cholera cases admitted to the adult ICU of a tertiary care hospital in South India, reflecting the disease’s clinical behaviour and outcomes in the current healthcare era.

Methodology

This retrospective study included all the patients admitted to the adult ICU at a tertiary care centre, in Bengaluru with acute gastroenteritis and subsequently diagnosed with cholera either by darting motility in the stool or culture positivity or both in the past one year and the data on clinical profile including demographic parameters, clinical presentation (number of episodes and nature of diarrhoea, and vomiting), hemodynamic parameters, and need for organ supports were collected. The severity of disease was assessed based on shock at presentation, graded by the shock index (heart rate/systolic blood pressure, HR/SBP), the presence and stage of acute kidney injury (AKI), and the severity of metabolic acidosis at presentation.

Results

A total of 20 adult patients diagnosed with cholera (16 cases diagnosed by culture positivity, and four cases only by darting motility in the stool hanging drop preparation) were included in the study. All patients presented with watery diarrhoea, with a median stool frequency of 10 per day (mean 12.6). Vomiting was present in 16/20 patients. The mean shock index at presentation was 1 ± 0.47. 15 (75%) of patients were in the 0.6-1.0 range, two (10%) between 1.0-1.4, and three (15%) had ≥1.4 shock index. Acute kidney injury was present in 11 (55%) of patients (Stage 1: four (20%), Stage 2: four (20%), Stage 3: three (15%). Metabolic acidosis was common: four (20%) were normal, seven (35%) were mild, eight (40%) were moderate, and one (5%) were severe. Stool hanging-drop was positive in all patients, and culture positivity was recorded in 16/20 (80%). All culture-positive isolates were sensitive to tetracycline. The mean intravenous fluid requirement was 13.8 L, and the median ICU stay was 48 hours (12-72). Vasopressor support was required in four (20%) patients for short durations. All patients survived to discharge, with no hospital mortality, with a median hospital stay of three days (two to six days).

Conclusion

The present study reinforces that cholera, though a historic disease, remains a disease of contemporary relevance. With early suspicion, aggressive rehydration, and early and appropriate pre-emptive antibiotic use, morbidity and mortality can be substantially minimised. This study highlights that, despite cholera being an ancient disease, the isolates are still sensitive to tetracycline, yet ongoing vigilance is essential given the emergence of resistant strains.

## Introduction

Cholera continues to remain a major global public health threat, with the World Health Organization (WHO) reporting more than 700,000 cases and over 1,600 deaths in 2023, marking one of the largest global resurgences of cholera cases in two decades. The trend persisted in 2024, with over 500,000 suspected and confirmed cases reported globally. Between 1st January and 17th August 2025, a total of 409,222 cholera and Acute Watery Diarrhoea (AWD) cases and 4,738 deaths were reported worldwide [1]. India remains an endemic country, with the Integrated Disease Surveillance Programme (IDSP) documenting multiple seasonal outbreaks each year. In 2024, more than 10,000 cases and 52 deaths were reported in India across states such as Karnataka, Kerala, Gujarat, Maharashtra, and Odisha [2]. 

This retrospective study aims to analyse the clinical and microbiological profile of patients diagnosed with cholera admitted to the adult intensive care unit (ICU) of a tertiary care hospital in Bangalore, South India, reflecting the disease’s behaviour and outcomes in the modern healthcare era. Bangalore, the information and technology capital of India and often referred to as the Silicon Valley of the country, harbours a diverse population, including many expatriates. The emergence of cholera cases in such a highly developed urban environment underscores the need for strengthening public health infrastructure, improving water, sanitation, and hygiene (WASH) systems, and enhancing surveillance mechanisms to prevent outbreaks. This study aims to determine the clinical and microbiological profile and disease severity of cholera among patients admitted to the adult ICU of a tertiary care hospital over a one-year period.

## Materials and methods

This is a retrospective study conducted between March 2024 and March 2025 in adult patients (>18 years) admitted to the Intensive Care Unit (ICU), Aster Whitefield Hospital, Bangalore, with acute gastroenteritis who were subsequently diagnosed with cholera. Institutional ethics committee approval was obtained before data collection for this study. Cholera was diagnosed based on stool culture positivity for *Vibrio cholerae* or darting motility on a hanging-drop stool examination or both.

For each patient, demographic details, presenting symptoms (including number and nature of diarrhoeal episodes and presence of vomiting), vital signs, and arterial blood gas (ABG) values at admission were collected. Documentation was completed for hemodynamic parameters, the need for organ support, total fluid requirements in the ICU, duration of ICU stay and hospital stay, and ICU/in-hospital mortality. Since outbreaks of cholera are not uncommon in Bangalore, the patients had received a single stat dose of oral doxycycline 300 mg as part of standard treatment for profuse watery diarrhoea at the time of presentation to the emergency department [3]. Disease severity was evaluated using the Shock Index (SI), severity of acute kidney injury, and severity of metabolic acidosis. SI was calculated as heart rate (HR) divided by systolic blood pressure (SBP). Normal SI was taken as <0.6. 0.6-1 was graded as mild, 1-1.4 was graded as moderate and more than 1.4 was graded as severe shock. Acute Kidney Injury (AKI) was diagnosed and staged according to the Kidney Disease: Improving Global Outcomes (KDIGO) criteria [4]. Baseline serum creatinine was defined as the most recent creatinine value recorded 8-365 days prior to ICU admission. If no baseline value was available, serum creatinine was estimated by back-calculation using the MDRD equation with an assumed eGFR of 75 mL/min/1.73m² [5]. The severity of metabolic acidosis at admission was classified based on arterial pH and bicarbonate (HCO₃⁻) levels and was graded by extrapolating the well-established diabetic ketoacidosis (DKA) classification system to general metabolic acidosis. A pH of 7.25-7.30 and/or bicarbonate 15-18 mmol/L was classified as mild acidosis, a pH between 7.00-7.24 and/ or bicarbonate levels of 10-15 was graded as moderate, and a pH of less than 7.00 and bicarbonate level of less than 10 was graded as severe [6]. 

All stool samples were cultured in TCBS (Thiosulfate Citrate Bile Salts Sucrose) agar media incubated at 37°C overnight. Microbiological evaluation was done by reviewing stool hanging drop preparation, culture and sensitivity results to determine the microbiological profile and antimicrobial susceptibility patterns of confirmed *Vibrio cholerae* isolates. Collected data were entered in Excel software and analysed using SPSS software, version 29.0.2.0 (IBM Corp., Armonk, NY). Descriptive statistics of normally distributed continuous variables were summarised as mean and standard deviation, while others were given as median and range. Categorical variables were given as counts and percentages.

## Results

A total of 20 adult patients diagnosed with cholera were included in the study. The mean age was 44.4 years (median 36 years, range 23-85); 11 (55%) were female, and nine (45%) were male. Most patients (70%) had no significant comorbidity; the remainder had conditions such as hypertension, diabetes, ischemic heart disease, or rheumatoid arthritis (Table 1).

**Table 1:** Baseline characteristics of patients

Parameter	Value (n = 20)
Age (years) Mean ± SD	44.4 ±18.75
Sex (M:F)	9:11
Heart Rate (/min) Mean ± SD	98.1±18.47
Systolic Blood Pressure (mmHg) Mean ± SD	104.1±21.88
Shock Index (Mean ± SD)	1 ± 0.47
Total Leukocyte Count (/mm³) Median (range)	13,777 (5770-26890)
Lactates (mmol/L) Median (Range)	2.6 (0.6-11.2)

All patients presented with watery diarrhoea, with a median stool frequency of 10 episodes per day (Mean 12.6). Vomiting was present in 16/20 patients. The mean heart rate at admission was 98.1 ± 18.47 beats/minute, and the mean systolic blood pressure was approximately 104 mmHg. The mean shock index was 1 ± 0.47 (mean±SD). Fifteen (75%) of patients were in the 0.6-1.0 range, two (10%) between 1.0-1.4, and three (15%) had a shock index ≥1.4.

Laboratory analysis showed a median total leucocyte count of 13,777/mm³ (5770-26890). Mean serum creatinine was 1.79 mg/dL (1.79 ±1.13). Acute kidney injury (AKI), classified using KDIGO criteria, was present in 11 (55%) of patients (Stage 1: four (20%), Stage 2: four (20%), Stage 3: three (15%). Metabolic acidosis was common: four (20%) had normal acid-base status, seven (35%) mild acidosis, eight (40%) moderate, and one (5%) severe (Table 2).

**Table 2:** Disease severity AKI: Acute kidney injury.

	No. of Patients (%)
Shock Index	
0.6 – 1.0	15 (75%)
1.0–1.4	2 (10%)
≥1.4	3 (15%)
Metabolic Acidosis	
Mild	7 (35%)
Moderate	8 (40%)
Severe	1 (5%)
Normal Acid–Base	4 (20%)
Acute Kidney Injury	
No AKI	9 (45%)
Stage 1	4 (20%)
Stage 2	4 (20%)
Stage 3	3 (15%)

Stool hanging-drop was positive in all patients, and culture positivity was recorded in 16/20 (80%). Tetracycline sensitivity was present in all culture-positive isolates. The mean intravenous fluid requirement was 13.8 ± 7.4 litres, and the median ICU stay was 48 hours. Vasopressor support was required in four (20%) patients for brief periods. All patients survived to discharge, with no in-hospital mortality (Table 3).

**Table 3:** Outcome parameters IV: Intravenous, ICU: Intensive care unit.

	Value
IV Fluid Volume (mL) Median (Range)	12,721 (3125-30446)
ICU Stay (hours) Median (Range)	48 (12-72)
Length of hospital Stay(days) Median (Range)	3 (2-6)
Vasopressor Use	4 (20%)
Discharged	20 (100%)

**Figure 1:**
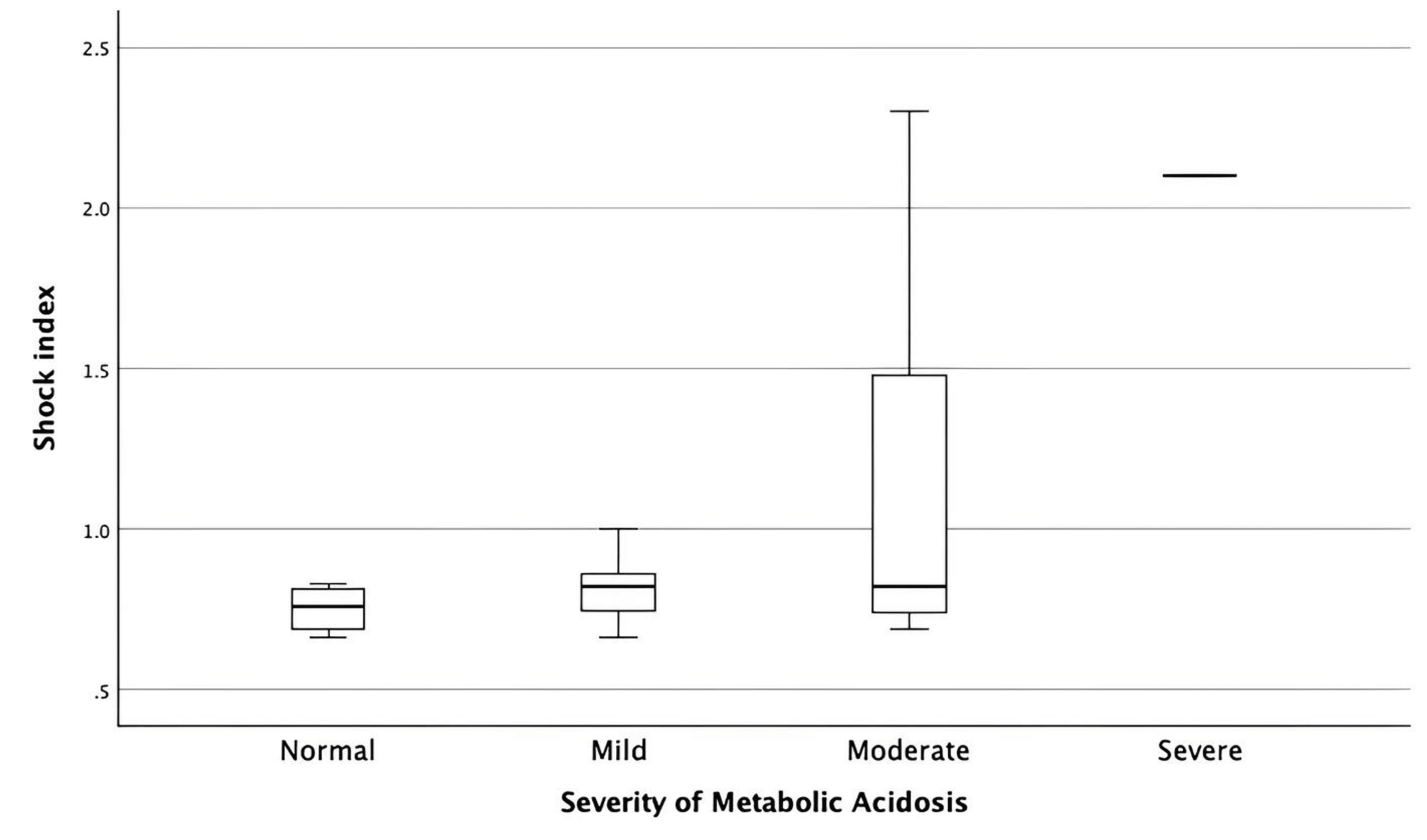
Shock index by severity of metabolic acidosis

Analysis of hemodynamic parameters showed a progressive rise in shock index with increasing severity of metabolic acidosis (Figure 1). Patients with normal or mild acidosis demonstrated shock index values largely within the normal to mildly elevated range, indicating relative hemodynamic stability. In contrast, those with moderate acidosis exhibited a broader distribution with several values exceeding 1.4, consistent with significant hypovolemia. The single patient with severe acidosis had the highest shock index, reflecting profound circulatory compromise.

**Figure 2:**
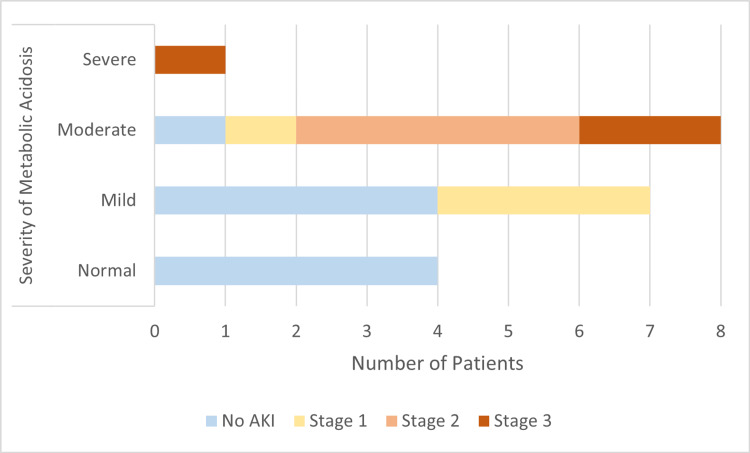
Severity of metabolic acidosis by AKI AKI: Acute kidney injury.

A similar trend was observed in the relationship between metabolic acidosis and acute kidney injury (Figure 2). AKI was uncommon in patients with normal acid-base status, whereas mild acidosis was associated with early-stage AKI. Moderate acidosis showed the highest burden of renal dysfunction, with patients distributed across all AKI stages. The lone patient with severe acidosis had Stage 3 AKI. These findings collectively demonstrate that worsening metabolic acidosis correlates with both higher shock index values and increasing severity of AKI, underscoring the impact of progressive dehydration and hypoperfusion in cholera.

## Discussion

Cholera is an acute infectious disease caused by toxigenic strains of *Vibrio cholerae*. Transmission occurs through the fecal-oral route, most commonly via ingestion of contaminated water or food. Despite major advances in prevention and treatment, cholera continues to present substantial public-health challenges. Conflicts, mass displacement, disasters from natural hazards, and climate change have intensified outbreaks predominantly in low- and lower-middle-income countries [7,8]. Cholera is diagnosed clinically based on acute, painless, profuse watery diarrhoea (“rice-water stools”). On laboratory evaluation, dark-field microscopy shows darting motility. Although this motility can also be seen with *Campylobacter jejuni* [9], in this study, the patient’s history of profuse watery stools with prevalence of local outbreaks makes *Vibrio cholerae* the most likely organism, and also, it is not pragmatic in our setting to attempt the culture of *Campylobacter jejuni*, which is a fastidious organism.

The gold standard for confirmation is stool culture. Stool or rectal swab samples are plated on Thiosulfate-Citrate-Bile-Salt Sucrose (TCBS) agar, where *V. cholerae* forms yellow sucrose-fermenting colonies, and inoculated into Alkaline Peptone Water (APW), an enrichment broth that enhances Vibrio growth before subculture onto solid media [10]. The present study provides an updated clinical and microbiological overview of cholera cases managed at a tertiary care corporate hospital in South India, offering insights into disease severity, complications such as AKI, and therapeutic outcomes. These findings are discussed in the context of national and global data to understand evolving trends in cholera epidemiology, management, and resistance patterns.

In this study, 20 cholera patients (16 patients diagnosed with culture positivity and four patients diagnosed on the basis of darting motility in the stool) were analysed in terms of severity and culture positivity of stools. Shock index (heart rate/systolic blood pressure) is a validated marker of hypovolaemic shock and adverse outcomes in various acute illnesses, but there is limited published evidence specifically evaluating its prognostic value in cholera. In this study, we used the shock index as a simple bedside indicator of haemodynamic compromise in patients with cholera, extrapolating from data in general hypovolaemic and emergency care populations [11]. In terms of shock index 15 (75%) of our patients were in the range of 0.6-1.0 (mild dehydration), two (10%) were between 1.0-1.4 (moderate dehydration), and three (15%) had ≥1.4 shock index reflecting severe dehydration. 

Metabolic acidosis in cholera is due to massive fluid and electrolyte losses from the gastrointestinal tract and impaired tissue perfusion. The degree of metabolic acidosis correlates with the severity of cholera. In a retrospective study carried out in 25 cholera patients admitted to the ICU in Mayotte (France), 16 patients had hypovolemia and severe metabolic acidosis requiring aggressive fluid replacement [12]. In our study, seven (35%) patients had mild metabolic acidosis, eight (40%) had moderate metabolic acidosis,1(5%) had severe metabolic acidosis, and four (20%) had normal acid-base balance. Mean lactate was 2.59 mmol/L. The degree of severity of metabolic acidosis was consistent with the amount of IV fluid requirement, with a mean IV fluid requirement of 13,819 mL.

AKI in cholera is typically prerenal, driven by reduced renal perfusion; however, if hypovolaemia is prolonged or inadequately corrected, it may progress to intrinsic renal injury. In a study by Vakrani and Nambakam, out of 55 patients, 12 had no AKI, and 43 (78)% patients had AKI, out of which 18 patients required renal replacement therapy [13]. In our study, out of the 20 patients analysed, nine (45%) did not have AKI, while 11 (55%) developed some degree of AKI. According to KDIGO classification, four (20%) had Stage 1, four (20%) had Stage 2, and three (15%) had Stage 3 AKI. None required renal replacement therapy.

Most cases were pre-renal and reversible with timely fluid replacement, highlighting that early correction of hypovolemia is pivotal in preventing progression to higher AKI stages. Notably, no cases of renal failure requiring dialysis were documented in our series, underscoring the effectiveness of our management protocols. These findings indicate a downward trend in AKI-related morbidity and support the role of early recognition and aggressive rehydration in reducing renal complications. In the current study, the clinical presentation remained typical of cholera, with profuse watery diarrhoea, vomiting, and severe dehydration. The disease severity appeared milder than reported in earlier decades, likely due to early presentation to medical facilities and early management before severe complications. Similar trends have been observed globally, where improved rehydration strategies and early antibiotic therapy have substantially reduced case fatality rates, often to below 1%.

Compared with earlier Indian series reporting mortality rates of more than 1% during outbreaks [14,15], our study demonstrated notably lower mortality, reflecting the impact of early presentation to medical facilities, prompt suspicion, aggressive rehydration, and antibiotic stewardship practices. Fluid resuscitation remains the cornerstone of cholera management. In our study, most patients received appropriate rehydration within the first hour of hospital arrival, with isotonic solutions guided by WHO protocols [16]. Early intervention was associated with rapid hemodynamic stabilization and favourable renal outcomes. This underscores the continuing relevance of simple yet effective measures such as oral rehydration solution (ORS) and intravenous Ringer’s lactate, which have been instrumental in the global decline of cholera mortality over the last two decades.

**Figure 3:**
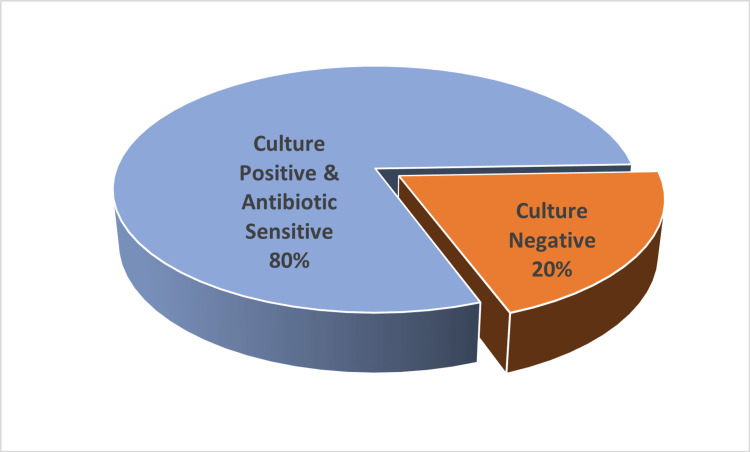
Culture positivity of cholera patients

The management of cholera has evolved remarkably over the past two centuries, transitioning from empirical remedies to evidence-based, physiology-driven therapy that has dramatically reduced mortality. In this study, out of 20 cases, 16 cases were culture positive (Figure 3). All the culture-positive strains were susceptible to tetracycline (16/16). Fifteen out of 16 patients were sensitive to ciprofloxacin, and one was having Intermediate sensitivity. Eleven out of 16 patients were sensitive to ampicillin, four patients had intermediate sensitivity, and one was resistant. Cefotaxime was tested only in six cases, and all were susceptible.

Microbiological analysis revealed that *Vibrio cholerae* isolates displayed varying susceptibility patterns, with emerging resistance noted to fluoroquinolones and certain macrolides, while tetracyclines remained largely effective. These findings are consistent with reports from national surveillance networks and WHO data highlighting rising multidrug resistance (MDR) among *V. cholerae* O1 El Tor strains. Strain identification was not done for these patients. The shifting sensitivity patterns underscore the need for continuous local antibiogram monitoring and rational antibiotic use. Antibiotic therapy is expected to shorten the volume and duration of diarrhoea and also reduce the duration of infectivity. Empirical therapy should be guided by institutional trends rather than historical choices. The role of antibiotic stewardship programs is critical in preventing further resistance development and ensuring therapeutic efficacy during future outbreaks.

Study limitations

This study has several inherent limitations. Firstly, its retrospective design may introduce bias related to incomplete documentation or the selection of more severe cases requiring hospitalisation. Secondly, being a single-centre study, the findings may not fully represent the epidemiological diversity of other regions. Thirdly, the sample size was modest, limiting statistical power for subgroup analyses, particularly for complications such as AKI and mortality predictors. 

Additionally, baseline renal function in some patients had to be estimated using the MDRD back-calculation method, which, while standardised, may not perfectly reflect true baseline values. Despite these limitations, the study provides valuable real-world data from a tertiary care context and contributes to the growing body of literature on contemporary cholera management. 

Lastly, four out of 20 were diagnosed with cholera based on stool positivity for darting motility (the remaining 16 were culture positive) in view of the prevalence of cholera outbreaks in our area. Culture or darting motility need not be positive in all cases of cholera. Although *Campylobacter jejuni *remains a differential diagnosis when darting motility is observed in stool samples, routine culture is impractical in our setting because it is a fastidious organism requiring specialized media and microaerophilic conditions. Furthermore, severe Campylobacter infections typically exhibit poor response to tetracycline therapy.

## Conclusions

The present study, done during a cholera outbreak in the city of Bengaluru, reinforces that cholera, though ancient, still remains a disease of contemporary relevance. With early suspicion, aggressive rehydration, and pre-emptive antibiotic use, the morbidity can be substantially minimized, and mortality can be prevented. This study also highlights that, despite cholera being an ancient disease, local isolates remain susceptible to tetracyclines. However, continued vigilance is required in view of emerging antimicrobial resistance.
